# Dynamics of Pneumococcal Acquisition and Carriage in Young Adults during Training in Confined Settings in Israel

**DOI:** 10.1371/journal.pone.0046491

**Published:** 2012-10-08

**Authors:** Hagai Levine, Ran D. Balicer, Salman Zarka, Tamar Sela, Vladislav Rozhavski, Daniel Cohen, Raid Kayouf, Ruhama Ambar, Nurith Porat, Ron Dagan

**Affiliations:** 1 Medical Corps, Israel Defence Force, Tel HaShomer, Israel; 2 Braun Hebrew University-Hadassah School of Public Health and Community Medicine, Jerusalem, Israel; 3 Faculty of Health Sciences, Ben-Gurion University of the Negev, Beer-Sheva, Israel; 4 School of Public Health, Haifa University, Haifa, Israel; 5 School of Public Health, Tel Aviv University, Tel Aviv, Israel; 6 Pediatric Infectious Disease Unit, Soroka University Medical Center, Beer-Sheva, Israel; Health Protection Agency, United Kingdom

## Abstract

**Background:**

Outbreaks and sporadic cases of pneumococcal illness occur among young adults in confined settings. Our aim was to characterize pneumococcal acquisition and carriage among healthy young adults in Israel during military training in confined settings.

**Methods:**

During the years 2007–2008, an observational longitudinal study was conducted in three cohorts of healthy soldiers, during a 7-month basic training period. Epidemiological data, oropharyngeal and nasopharyngeal cultures were sampled on 5 occasions: before and 3, 6, 12 and 24 weeks after start of training. Samples were processed within 2–18 hours. Relatedness of isolates was investigated by capsular typing of all isolates and pulsed-field gel electrophoresis to determine acquisition and transmission. Carriage and acquisition patterns were analyzed and multivariable logistic regression analysis was performed to assess the impact of time on acquisition after mixing, controlling for other covariates.

**Results:**

Pneumococci were recovered on 202 of 1872 visits among 742 individuals, including 40 different serotypes. Mean carriage prevalence increased in all visits following training initiation. Acquisition during training was high, as 36.9% of individuals acquired pneumococci at least once during training, and for almost one fourth of the whole population this occurred during the first 6 weeks. Significant clustering was noted. Sharing drinking glass/bottle was found to be a significant and common risk factor for pneumococcal acquisition.

**Conclusions:**

Pneumococcal acquisition is highly frequent when young adults live in close contact in confined settings, especially early after mixing.

## Introduction

*Streptococcus pneumoniae* is the most common bacterial etiology of community-acquired pneumonia in all ages, and can cause outbreaks in closed settings [1], [2], [3]. Person-to-person transmission is common [1]. Upper respiratory tract carriage is an important precursor of pneumococcal disease and is increased under circumstances that facilitate transmission, such as overcrowding [4]. Most data on pneumococcal transmission derive from studies conducted in children and less often in their caregivers [5], [6], [7], [8], [9], but transmission patterns and dynamics among healthy young adults are less known [5]. A recent outbreak of severe pneumococcal illness in an Israeli army training base [10] led to undertake a study to characterize pneumococcal carriage prevalence, acquisition and dynamics among healthy young recruits before and during military training in confined settings. Our hypothesis was that during military training, especially soon after mixing, a significant rise in pneumococcal carriage rates will be common due to frequent acquisitions, as previously found for other pathogens [11], [12].

## Methods

### Study design, setting and population

This observational prospective longitudinal study was conducted in three consecutive cohorts, recruited to one Israel Defense Force (IDF) training base in March, July and November 2007. All subjects were examined before mixing and were followed during the 7-month basic training period. Epidemiological and medical data (socio-economic variables, morbidity and health behavior data) and oropharyngeal (OP) and nasopharyngeal (NP) pneumococcal cultures were sampled on 5 occasions: before start of training (visit 1) and 3, 6, 12 and 24 weeks after start of training (visits 2–5, respectively). The trainees were divided into smaller companies two weeks after initiation of training. Selection of studied companies for acquisition during training was based on the highest number of potential participants, up to 140 participants per cohort, in accordance with study protocol and sample size calculations. Subjects with any known underlying chronic medical condition were excluded from basic training and hence from the study. Training was characterized by extensive physical activities and stressful conditions.

During the first 3 months of training, soldiers lived mostly in functional, air-conditioned rooms (12–16 per room); during the late 3 months soldiers lived mostly in the field (2 per tent). Contacts within each company were frequent, while contacts between companies from the same cohort were less frequent and contacts between cohorts were rare. Participants of the November and August cohorts were vaccinated against influenza in November. Participants had not been previously vaccinated against pneumococcus.

The study was performed before the introduction of routine pneumococcal childhood vaccination in Israel in July 2009. Each subject signed an informed consent before being recruited to the study. The study was approved by the Medical Corps Ethics Board of the IDF.

### Variables and data sources

Only proper samples obtained from both NP and OP were considered valid. Valid sampling on the first visit, before training, was a prerequisite for further analysis. Carriage prevalence was defined as proportion of individuals with NP and/or OP *S. pneumoniae* isolates divided by the appropriate denominator of valid samplings. Acquisition was defined as isolation of *S. pneumoniae* serotype that was not isolated in any previous visit. For calculations of acquisition, a sampling was counted only if proper sampling of both NP and OP was taken in all previous visits. Reporting answer to frequency of sharing drinking glass/bottle was grouped to frequent sharing (‘always/usually’) and non-frequent sharing (‘half of the time/occasionally/never’). Seasons were grouped as 3 periods, in accordance with the 3 recruitment periods, March–June, July–October and November–February.

### Laboratory procedures

The sampling was conducted by a trained team of healthcare workers according to a defined protocol. Samples were inoculated onto a transport medium, cultured within 2–18 hours and processed for identification and serotyping as described previously [13]. Serotyping was tested by means of the quellung reaction using antisera provided by Statens Serum Institute of Copenhagen, Denmark [14].

Pulsed-field gel electrophoresis (PFGE) was carried as described previously [15] for all isolates in the study, except when only one isolate was found for this serotype. PFGE was performed to identify clustering within cohorts, differentiate continued carriage from acquisition in the individual, and examine similarities between NP and OP isolates in the same person at the same time. Serotype 6A was differentiated from serotype 6C [16]. Clones were defined by the same serotype and similar PFGE pattern (up to 6 bands difference). Clonal group was defined as 2 or more isolates of a specific clone found in the entire study. In the case of multiple isolates of identical characteristics from a given subject, only the first isolate was considered for the calculation of acquisition or clonal group.

### Statistical methods

Statistical analysis was performed by SAS software, version 9.2 (SAS Institute Inc., Cary, NC, USA). Proportions of carriage prevalence and acquisition rates were calculated for each cohort and visit. 95% confidence intervals (CIs) for prevalence and acquisition rates were calculated through binomial distribution. Univariable analysis for risk factors of pneumococcal prevalence and acquisition was performed by Chi-square test. *P*<.05 was considered to be statistically significant. We performed multivariable logistic regression analysis evaluating risk of carriage after versus before mixing and risk of acquisition by time during training controlled for possible confounders identified by univariable analysis. Analysis for acquisition controlled also for soldier companies, which was not possible for prevalence analysis due to subdivision to companies 2 weeks after recruitment. The multivariable risk factors analysis for longitudinal dichotomy variables were assessed by repeated measures technique for binary response. Two methods for longitudinal acquisition analysis were assessed: balanced (participants in all visits only) and unbalanced (all participants in ≥2 visits, contribution until loss to follow-up). For regular dichotomy responses, logistic regression was used. The results of both multivariable methods were presented by odds ratio (OR) and 95% CI. Cluster unit was defined as having more than 50% of clones from the same clonal group in one of the three cohorts. For each clonal group, chances of being a cluster unit were calculated under the assumption that the probability of isolates occurring in each cohort is constant and equals one third. The expected number of cluster units was calculated by multiplying the observed number of clonal groups with the chances of being a cluster unit. Chances of having the observed number of cluster units or more for each size of clonal group were calculated using binomial distribution.

## Results

**Figure 1:**
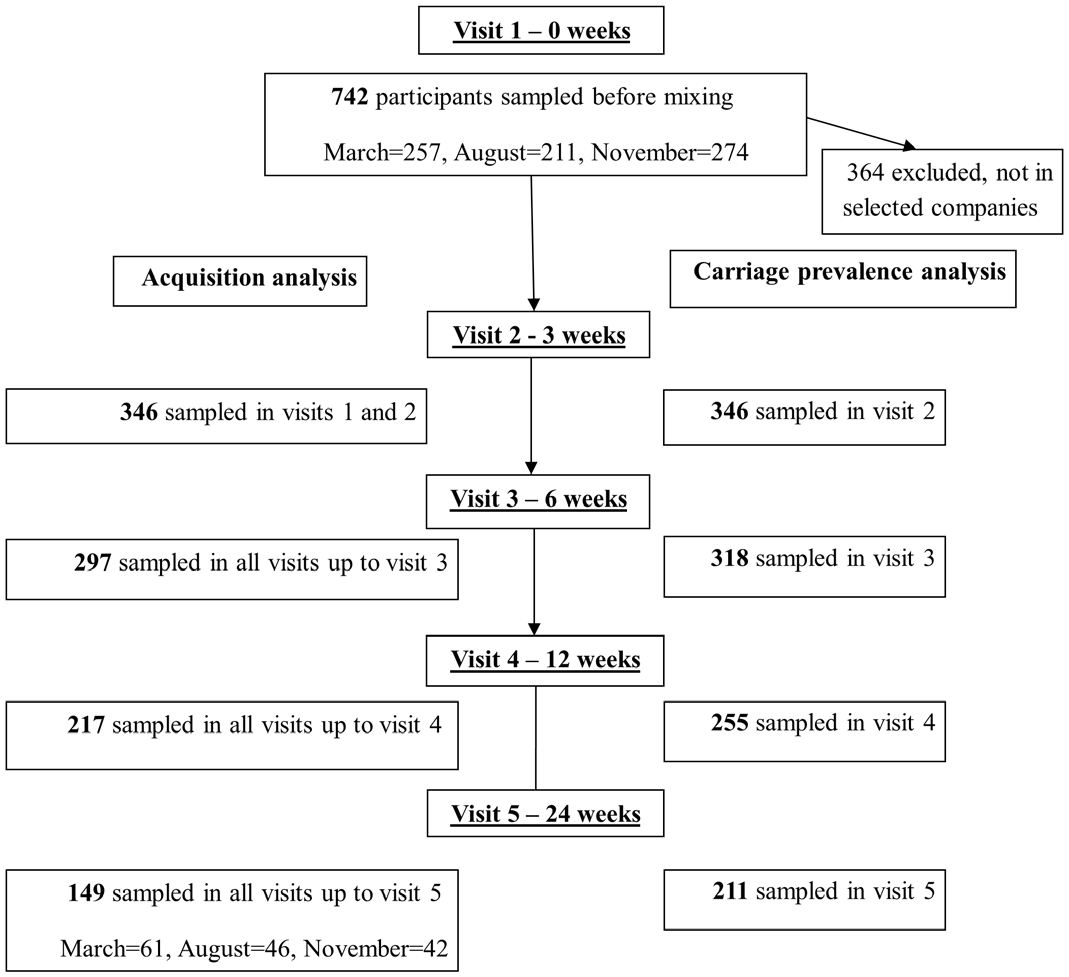
Flow chart of 742 participants in pneumococcal carriage study, for acquisition and for carriage prevalence analyses.

Of 1340 male recruits invited to participate in the study, 776 (57.9%) agreed to participate, of whom 742 (95.6%) underwent proper sampling in the first visit. The flow chart of participants analyzed for acquisition and carriage prevalence analysis is shown in **Figure 1**. Socio-economic and health behavior characteristics, as well as pneumococcal carriage before training, were described previously [17]. There were no significant differences in any of the socio-economic and health behavior characteristics or pneumococcal carriage before start of training between the 346 participants who were sampled in the second visit and the other 396 participants, not selected for the longitudinal study, except higher household crowding among participants in the second visit. Prevalence tended to be higher but did not differ significantly in none of the visits between those who were sampled in all 5 visits and those missing ≥1 samples. A significant trend (*P*<.001) of increase in sharing of glass/bottle during training was observed. None of the study participants required medical hospitalization due to pneumonia or pneumococcal related illness during the study period.

### *S. pneumoniae* carriage prevalence

**Figure 2:**
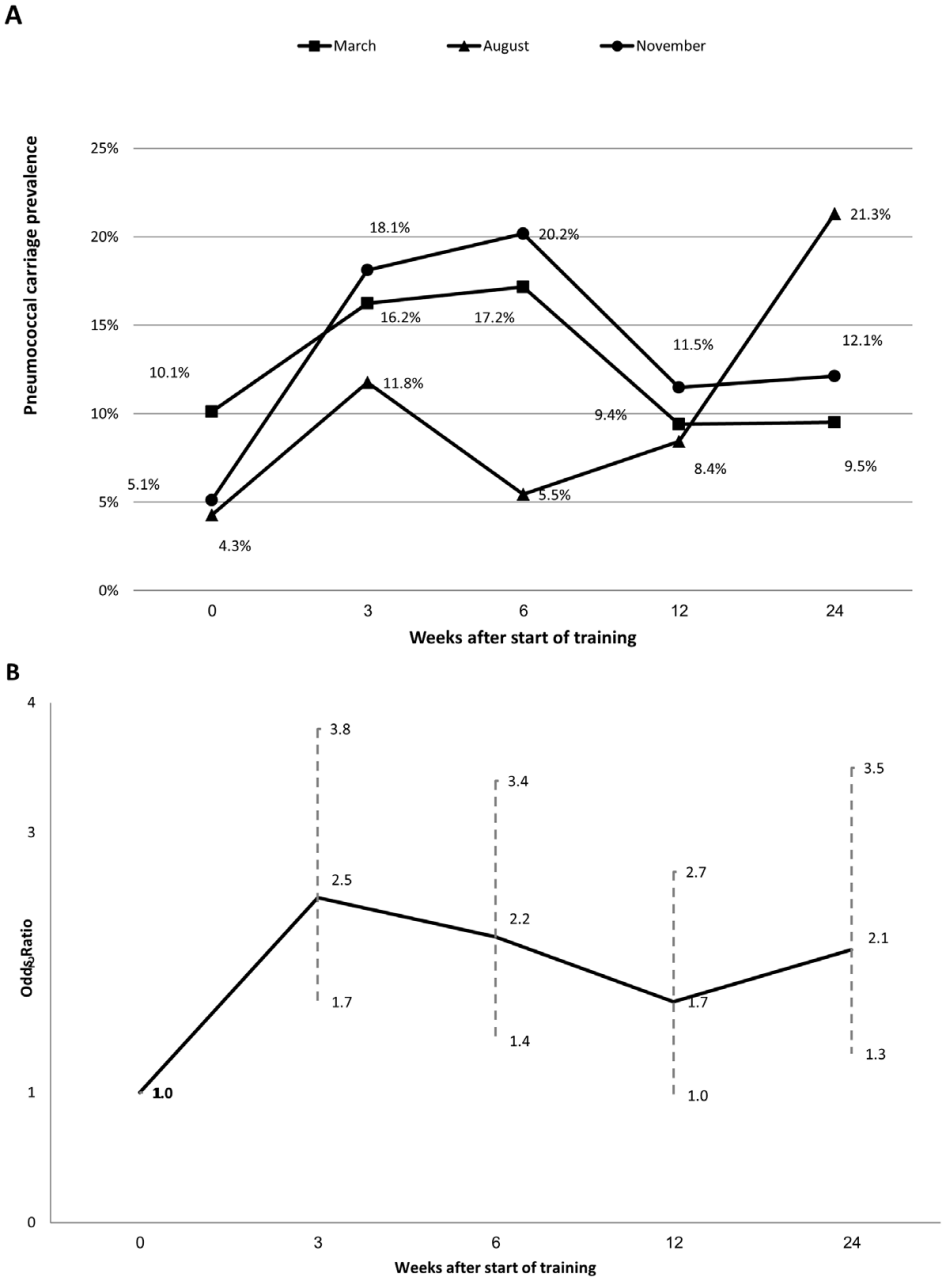
A. Pneumococcal carriage prevalence by cohort and weeks since start of training. B. Odds ratio and 95% confidence interval for pneumococcal carriage prevalence during training compared to first visit, adjusted for season and frequency of sharing drinking glass.

Pneumococci were recovered from 202 of 1872 (10.8%, 95% CI 9.4–12.3%) samples from 742 individuals, including 40 different serotypes. The most common serotypes (and their proportion of all isolates) were Omni Neg (15%), 3 (8%) 19F (6%), 6A, 6C, 15B, 34 (5% each), constituting together 49% of all isolates. Of 202 positive samples, 118 (58.4%), 42 (23.8%) and 36 (17.8%) were from NP alone, OP alone and both NP and OP, respectively. In the 36 subjects from whom both NP and OP yielded an isolate, serotypes were identical in 27 (75%) and different in 9 (25%). In all three cohorts, carriage prevalence tested 3 weeks after mixing increased, most notably in the November cohort, in which the prevalence increased more than 3-fold (**Figure 2A**). In univariable analysis, time since start of training, high frequency of sharing drinking glass/bottle and seasonality were risk factors for carriage. After adjusting for frequency of sharing drinking glass/bottle and seasonality, carriage after coming into contact with each other remained a significant risk factor compared to pre-mixing period (OR = 2.10, 95% CI 1.49–2.98, *P* = .001). Risk was higher in any of the visits post-mixing vs. pre-mixing with a highest trend 3 weeks after start of training (**Figure 2B**). High frequency of sharing drinking glass (OR = 1.86, 95% CI 1.34–2.57, *P*<.001), and season were significant risk factors; with higher risk in March–June (OR = 1.86, 95% CI 1.21–2.86, *P* = .003) and in November–February (OR = 1.81, 95% CI 1.15–2.83, *P* = .007) compared to July–October.

### *S. pneumoniae* carriage acquisition

**Figure 3:**
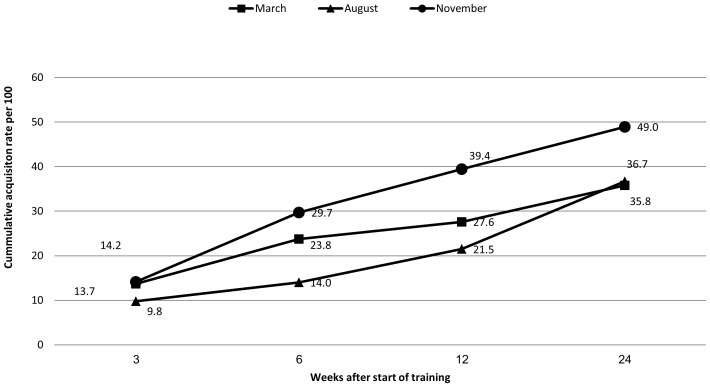
Pneumococcal cumulative acquisition rate per 100 participants by cohort and weeks since start of training.

Cumulative acquisition rates (per 100 participants) during the study period were higher in the November cohort compared to August and March cohorts (*P*<.001 for each, **Figure 3**). Acquisition was highest in the early period, 3 weeks after mixing in a confined setting (12.7%, 95% CI 9.2–16.2%). Of 149 who participated in all visits, in 55 (36.9%) at least one acquisition was detected in any of the post-mixing visits; one new acquisition was demonstrated in 44 (29.5%) subjects, 2 in 10 (6.7%) and 3 in one subject.

We chose to display the results of the multivariable analysis for risk of pneumococcal acquisition during training by time since mixing in 2 separate models (**Table 1**): A balanced model in which only those who were examined in all 5 visits are displayed and an unbalanced model in which all contributions until loss to follow-up are displayed. The highest risk of acquisition compared to all visits was observed in the 3 weeks that followed their first mixing in both analyses but did not reach statistical significance in most comparisons. However, in the balanced model, risk was significantly lower in all other visits combined compared with the 3-week visit (OR = 0.50, 95% CI 0.28–0.87, *P* = .015). Frequent glass/bottle sharing (reported in each visit) was significantly associated with pneumococcal acquisition in the balanced model (OR = 2.32, 95% CI 1.25–4.33, *P* = .009) but did not reach statistical significance in the unbalanced model (*P* = .07). Risk was highest in November–February and lowest in July–October, significant for direct comparison in the unbalanced (*P* = .01) but not reaching significance in the balanced model (*P* = .052). Adjusted mean acquisition rate per visit was 9.8% (95% CI 7.5–12.7%) for the unbalanced and 10.0% (95% CI 7.1–14.2%) for the balanced model.

**Table 1:** Timing of acquisition of pneumococci during training in confined setting in Israel, identified by multivariable repeated measures analysis^a^.

	Unbalanced model^b^		Balanced model (only complete follow-up)^c^	
	P value	Odds Ratio (95% CI)	P value	Odds Ratio (95% CI)
Time since start of training	.			
3 weeks	.53	1.0	.11	1.0
6 weeks	.22	0.73 (0.40–1.34)	.11	0.57 (0.25–1.32)
12 weeks	.25	0.68 (0.30–1.52)	.04	0.42 (0.15–1.14)
24 weeks	.50	0.80 (0.37–1.73)	.07	0.52 (0.22–1.23)
6, 12, 24 weeks combined	.16	0.73 (0.47–1.14)	0.015	0.50 (0.28–0.87)

a Controlling for sharing drinking glass frequency, company and three seasons.

b 1003 observations included n unbalanced model, including contribution until loss to follow-up.

c 593 observations included in balanced model, i.e. including only those who were sampled in all 5 visits.

### Pneumococcal serotypes and clustering

Serotyping and PFGE were performed to investigate clustering by cohorts. Overall, 32 clonal groups of 2 or more clones in the entire study were found. Cluster unit was defined as having >50% of clones from the same clonal group in one of the three cohorts. Highly significant clustering was observed (**Table 2**). Twenty four cluster units were observed, significantly more than the expected 8 cluster units (*P*<.001). Clustering was still significant (*P*<.001) when the definitions of the needed >50% of the clones in one cohort was replaced by >67%. The largest cluster units of 4 or more clones were composed of serotypes 15B, 3, 37, 34, 6C, 6A and 15A.

**Table 2:** Observed and expected cluster units of pneumococcal clones in three cohorts of trainees, Israel, 2007.

Distribution of clonal groups by number of isolates per group		Presence of cluster units^a^ in any of the 3 cohorts		Number of cluster units in the current study		
Number of isolates per group	Observed number of groups	Minimal number needed for cluster unit	*P* value for being a true cluster unit for each clonal group	Expected^b^	Observed	*P* value of the observed number of cluster units to be beyond simple chance
2	14	2	.33	4.67	8	.058
3	8	2	.33	2.67	8	<.001
4	5	3	.11	0.56	3	.012
5	1	3	.09	0.09	1	.09^c^
7	3	4	.021	0.06	3	<.001
9	1	5	.005	0.005	1	.005
**Overall**	**32**			**8.06**	**24**	**<.001**

a A cluster unit was defined by the presence of >50% of identified clones in one of the 3 cohorts.

b Expected number of cluster units in the current study for each cluster unit size was derived by multiplying the observed number of groups with the same clone and the same size by the *P*-value for being a true cluster unit.

c For clonal group of 5, if minimal number needed for cluster unit is defined as ≥4 (as was the observed cluster unit) then *P* value is .037.

On all occurrences of multiple isolation of same serotype from same individual, a single clone was found, however, in two cases the same serotype was isolated both from NP and OP in the same individual on the same occasion but differed by PFGE. For quality assurance all pairs of different serotypes recovered from NP and OP were examined by PFGE and in all cases the isolates differed by PFGE. All 26 isolates first identified from 15 persons as 6A were re-examined to differentiate them from 6C. Thirteen isolates in 7 persons were identified as 6C.

## Discussion

To the best of our knowledge, acquisition of pneumococci in confined settings of young adults without contact with children was not previously investigated, except in the context of an outbreak investigation. In the current study, soon after mixing of recruits for training in a confined setting, the mean pneumococcal carriage prevalence increased significantly. Despite the fact that the subjects were not sampled frequently, a high rate of new acquisitions was detected during the first 24 weeks following their mixing, as approximately one third of the subjects acquired a new *S. pneumoniae* isolate.

The acquisition risk was highest during the first 3 weeks of training although the risk magnitude differed by season of recruitment. Significant clustering pattern in cohorts was observed. Sharing drinking glass/bottle was found for the first time in our study to be a significant and common risk factor for pneumococcal acquisition, at least for young adults.

The observed carriage during training in confined settings was relatively high compared to baseline prevalence. Previous studies on carriage of *S. pneumoniae* among young adults have focused on caregivers of young children. In a family study conducted in the UK, carriage prevalence in individuals >18 years old was 8% [6]; the prevalence was 4–15% in studies conducted in Israel [9], [18]. Relatively high carriage prevalence of 15.7% was found among trainees in the US Army [2] and carriage >20% was reported during outbreaks [10], [19]. Significant OP carriage was found in our study as one fourth of isolations would have been missed if only NP had been taken. This is similar to findings in other studies among young adults [18], [20]

Possible explanations for the increased risk of carriage during training and especially after first mixing in confined setting include multiple exposures to numerous individuals, especially due to low hygiene and sharing saliva through glasses, and crowded settings. Our findings of marked, early and fast acquisition and spread of *S. pneumoniae* immediately following the initiation of training of the recruits, help explain previous reports of outbreaks caused by known virulent pneumococcal strains such as serotype 1, 5 or 12F in confined settings in previously healthy young individuals [3], [10], [21]. However, as found in our study, in most instances, carriage and spread of pneumococci, even if extensive, is mainly asymptomatic and does not lead to outbreaks.

We detected a clear cohort-specific clustering of *S. pneumoniae* clones implying that each cohort has its own microenvironment. A similar finding was previously found among children attending day care centers in Israel and elsewhere [22], [23], [24]. The fact that such clustering was evident despite our design which did not allow frequent samplings, suggests an extensive exchange of *S. pneumoniae* clones among trainees.

Sharing drinking utensils is a common practice among young adults in Israel, and half of participants were frequently sharing drinking glass/bottle before recruitment. This became even more common during training as two-thirds of the participants reported sharing at the end of training. Our previous report showed that frequent sharing of drinking glass/bottle was a common, strong and independent risk factor for pneumococcal carriage prevalence [17]. The current report extends this finding to pneumococcal acquisition as well. These results are unlikely to be associated with general hygiene practices, since no association was found between hand washing frequency and pneumococcal carriage and not confounded by other known risk factors such as smoking or seasonality as those were controlled for. Since glass/bottle sharing increased during training, we believe that educating the recruits to reduce frequency of sharing may help reduce the spread of pneumococci among trainees.

Our study suggests higher carriage and acquisition of pneumococci in the winter months compared to the summer months, as was previously found for children. [25], [26], [27] Transmission of respiratory pathogens and that of *S. pneumoniae* in particular, may be enhanced during the winter months because of behavioral change (such as increased crowding), lack of adequate ventilation or viral infections such as influenza. Our finding, together with the occurrence of previous pneumococcal outbreaks among recruits in the IDF in the winter months, [10] point to the need of special awareness during this high risk period as well as further research to understand the exact mechanism of higher transmission in the winter months.

The serotypes isolated from NP/OP were diverse. When counted once for each appearance, only 14%, and 24% of all acquired isolates were caused by the 10-valent and 13-valent vaccine serotypes included in the pneumococcal conjugate vaccines (PCV10 and PCV13, respectively) and only 39% were included in the 23-valent non-conjugate polysaccharide vaccine (PPV23). Thus, when considering acquisition of carriage, pneumococcal conjugate vaccines may not be the best choice as a preventive measure. However, many of the carried serotypes are relatively not virulent, while outbreaks in military corps and closed community may be caused by some of the more virulent serotypes, such as serotypes 1, 5, or 7F or 12F. [3], [10], [21], [28] The routine use of PPV23 in army units is debated. [28] Other measures such as prophylactic antibiotics are costly, have side effect and promote antibiotic resistance.

Our study has several limitations. First, the sample size was large enough for an analysis of common and strong risk factors but did not enable to rule out other possible risk factors such as smoking. This relatively small sample size did not allow investigation of clustering by companies. Second, the relatively low frequency of 5 samplings in a 24 week-period leads to a probable underestimate of the true rate, likely to be significantly higher. Third, only 43% of the selected subjects were followed during all visits, limiting our power to identify risk factors and clustering pattern. However, a comparison of available socio-economic characteristics and pneumococcal carriage did not reveal major differences between the groups and there is no reason to suspect different clustering pattern or associations between risk factors and carriage. This is supported by the results of the risk factors model which were overall similar for the entire group and only for those who completed follow-up. Fourth, although we controlled for several confounders, we did not control for other possible confounders such as intimate kissing or attendance at pubs/bars previously found to be associated with other respiratory tract pathogens [29].

In conclusion, acquisition and spread of pneumococci during the crowded gathering of young adults in confined settings is common, with the highest risk occurring soon after mixing. These findings are used in determining public health policy in the Israeli army and support applying special emphasis on prevention and surveillance of pneumococci and other pathogens during the high risk first period of young adults' meeting in a crowded environment.

## References

[1] Moore M, Qazi S (2008) Pneumococcal pneumonia. In: Heyman D, editor. Control of Communicable Diseases Manual. 19th Edition. Washington, DC: American Public Health Association, pp 471–476.

[2] Crum NF, Wallace MR, Lamb CR, Conlin AM, Amundson DE, et al (2003) Halting a pneumococcal pneumonia outbreak among United States Marine Corps trainees. Am J Prev Med 25: 107–111. 10.1016/s0749-3797(03)00114-4 12880877

[3] Dagan R, Gradstein S, Belmaker I, Porat N, Siton Y, et al (2000) An outbreak of *Streptococcus pneumoniae* serotype 1 in a closed community in southern Israel. Clin Infect Dis 30: 319–321. 10671335 10.1086/313645

[4] Bogaert D, De Groot R, Hermans PW (2004) *Streptococcus pneumoniae* colonisation: the key to pneumococcal disease. Lancet Infect Dis 4: 144–154. 14998500 10.1016/S1473-3099(04)00938-7

[5] Garcia-Rodriguez JA, Fresnadillo Martinez MJ (2002) Dynamics of nasopharyngeal colonization by potential respiratory pathogens. J Antimicrob Chemother 50 Suppl S2: 59–73. 10.1093/jac/dkf506 12556435

[6] Hussain M, Melegaro A, Pebody RG, George R, Edmunds WJ, et al (2005) A longitudinal household study of *Streptococcus pneumoniae* nasopharyngeal carriage in a UK setting. Epidemiol Infect 133: 891–898. 16181510 10.1017/S0950268805004012 PMC2870321

[7] Melegaro A, Gay NJ, Medley GF (2004) Estimating the transmission parameters of pneumococcal carriage in households. Epidemiol Infect 132: 433–441. 15188713 10.1017/s0950268804001980 PMC2870123

[8] Greenberg D, Givon-Lavi N, Broides A, Blancovich I, Peled N, et al (2006) The contribution of smoking and exposure to tobacco smoke to *Streptococcus pneumoniae* and *Haemophilus influenzae* carriage in children and their mothers. Clin Infect Dis 42: 897–903. 16511750 10.1086/500935

[9] Regev-Yochay G, Raz M, Dagan R, Porat N, Shainberg B, et al (2004) Nasopharyngeal carriage of *Streptococcus pneumoniae* by adults and children in community and family settings. Clin Infect Dis 38: 632–639. 14986245 10.1086/381547

[10] Balicer RD, Zarka S, Levine H, Klement E, Sela T, et al (2010) Control of *Streptococcus pneumoniae* serotype 5 epidemic of severe pneumonia among young army recruits by mass antibiotic treatment and vaccination. Vaccine 28: 5591–5596. 20599301 10.1016/j.vaccine.2010.06.031 PMC7126119

[11] Block C, Gdalevich M, Buber R, Ashkenazi I, Ashkenazi S, et al (1999) Factors associated with pharyngeal carriage of *Neisseria meningitidis* among Israel Defense Force personnel at the end of their compulsory service. Epidemiol Infect 122: 51–57. 10098785 10.1017/s0950268898001769 PMC2809587

[12] Jousimies-Somer HR, Savolainen S, Ylikoski JS (1989) Comparison of the nasal bacterial floras in two groups of healthy subjects and in patients with acute maxillary sinusitis. J Clin Microbiol 27: 2736–2743. 2592539 10.1128/jcm.27.12.2736-2743.1989 PMC267119

[13] Dagan R, Givon-Lavi N, Fraser D, Lipsitch M, Siber GR, et al (2005) Serum serotype-specific pneumococcal anticapsular immunoglobulin G concentrations after immunization with a 9-valent conjugate pneumococcal vaccine correlate with nasopharyngeal acquisition of pneumococcus. J Infect Dis 192: 367–376. 15995949 10.1086/431679

[14] Austrian R (1976) The quellung reaction, a neglected microbiologic technique. Mt Sinai J Med 43: 699–709. 13297

[15] Porat N, Barkai G, Jacobs MR, Trefler R, Dagan R (2004) Four antibiotic resistant *S. pneumoniae* clones unrelated to the pneumococcal conjugate vaccine serotypes, including 2 new serotypes, causing acute otitis media in southern Israel. J Infect Dis 189: 385–392. 14745695 10.1086/381183

[16] Park IH, Park S, Hollingshead SK, Nahm MH (2007) Genetic basis for the new pneumococcal serotype, 6C. Infect Immun 75: 4482–4489. 17576753 10.1128/IAI.00510-07 PMC1951153

[17] Levine H, Zarka S, Dagan R, Sela T, Rozhavski V, et al (2012) Transmission of *Streptococcus pneumoniae* in adults may occur through saliva. Epidemiol Infect 140: 561–565. 21676361 10.1017/S0950268811000884

[18] Greenberg D, Broides A, Blancovich I, Peled N, Givon-Lavi N, et al (2004) Relative importance of nasopharyngeal versus oropharyngeal sampling for isolation of *Streptococcus pneumoniae* and *Haemophilus influenzae* from healthy and sick individuals varies with age. J Clin Microbiol 42: 4604–4609. 15472316 10.1128/JCM.42.10.4604-4609.2004 PMC522367

[19] Musher DM, Groover JE, Reichler MR, Riedo FX, Schwartz B, et al (1997) Emergence of antibody to capsular polysaccharides of *Streptococcus pneumoniae* during outbreaks of pneumonia: association with nasopharyngeal colonization. Clin Infect Dis 24: 441–446. 9114197 10.1093/clinids/24.3.441

[20] Watt JP, O'Brien KL, Katz S, Bronsdon MA, Elliott J, et al (2004) Nasopharyngeal versus oropharyngeal sampling for detection of pneumococcal carriage in adults. J Clin Microbiol 42: 4974–4976. 15528682 10.1128/JCM.42.11.4974-4976.2004 PMC525247

[21] Hoge CW, Reichler MR, Dominguez EA, Bremer JC, Mastro TD, et al (1994) An epidemic of pneumococcal disease in an overcrowded, inadequately ventilated jail. N Engl J Med 331: 643–648. 8052273 10.1056/NEJM199409083311004

[22] Givon-Lavi N, Dagan R, Fraser D, Yagupsky P, Porat N (1999) Marked differences in pneumococcal carriage and resistance patterns between day care centers located within a small area. Clin Infect Dis 29: 1274–1280. 10524975 10.1086/313465

[23] Leino T, Hoti F, Syrjanen R, Tanskanen A, Auranen K (2008) Clustering of serotypes in a longitudinal study of *Streptococcus pneumoniae* carriage in three day care centres. BMC Infect Dis 8: 173. 19116005 10.1186/1471-2334-8-173 PMC2639357

[24] Kellner JD, McGeer A, Cetron MS, Low DE, Butler JC, et al (1998) The use of *Streptococcus pneumoniae* nasopharyngeal isolates from healthy children to predict features of invasive disease. Pediatr Infect Dis J 17: 279–286. 9576381 10.1097/00006454-199804000-00004

[25] Yagupsky P, Porat N, Fraser D, Prajgrod F, Merires M, et al (1998) Acquisition, carriage, and transmission of pneumococci with decreased antibiotic susceptibility in young children attending a day care facility in southern Israel. J Infect Dis 177: 1003–1012. 9534975 10.1086/515239

[26] Gray BM, Converse GM 3rd, Dillon HC Jr (1980) Epidemiologic studies of *Streptococcus pneumoniae* in infants: acquisition, carriage, and infection during the first 24 months of life. J Infect Dis 142: 923–933. 7462701 10.1093/infdis/142.6.923

[27] Darboe MK, Fulford AJ, Secka O, Prentice AM (2010) The dynamics of nasopharyngeal *Streptococcus pneumoniae* carriage among rural Gambian mother-infant pairs. BMC Infect Dis 10: 195. 20602782 10.1186/1471-2334-10-195 PMC2910019

[28] Dawood FS, Ambrose JF, Russell BP, Hawksworth AW, Winchell JM, et al (2011) Outbreak of pneumonia in the setting of fatal pneumococcal meningitis among US Army trainees: potential role of *Chlamydia pneumoniae* infection. BMC Infect Dis 11: 157. 21635754 10.1186/1471-2334-11-157 PMC3121612

[29] MacLennan J, Kafatos G, Neal K, Andrews N, Cameron JC, et al (2006) Social behavior and meningococcal carriage in British teenagers. Emerg Infect Dis 12: 950–957. 16707051 10.3201/eid1206.051297 PMC3373034

